# AI Can Be a Powerful Social Innovation for Public Health if Community Engagement Is at the Core

**DOI:** 10.2196/68198

**Published:** 2025-01-22

**Authors:** Alessandra N Bazzano, Andrea Mantsios, Nicholas Mattei, Michael R Kosorok, Aron Culotta

**Affiliations:** 1 Department of Maternal and Child Health Gillings School of Global Public Health University of North Carolina at Chapel Hill Chapel Hill, NC United States; 2 Center for Community-Engaged Artificial Intelligence School of Science & Engineering Tulane University New Orleans, LA United States; 3 Public Health Innovation and Action New York, NY United States; 4 Department of Computer Science School of Science & Engineering Tulane University New Orleans, LA United States; 5 Department of Biostatistics Gillings School of Global Public Health University of North Carolina at Chapel Hill Chapel Hill, NC United States; 6 Gillings Center for Artificial Intelligence and Public Health Gillings School of Global Public Health University of North Carolina at Chapel Hill Chapel Hill, NC United States

**Keywords:** Artificial Intelligence, Generative Artificial Intelligence, Citizen Science, Community Participation, Innovation Diffusion

## Abstract

There is a critical need for community engagement in the process of adopting artificial intelligence (AI) technologies in public health. Public health practitioners and researchers have historically innovated in areas like vaccination and sanitation but have been slower in adopting emerging technologies such as generative AI. However, with increasingly complex funding, programming, and research requirements, the field now faces a pivotal moment to enhance its agility and responsiveness to evolving health challenges. Participatory methods and community engagement are key components of many current public health programs and research. The field of public health is well positioned to ensure community engagement is part of AI technologies applied to population health issues. Without such engagement, the adoption of these technologies in public health may exclude significant portions of the population, particularly those with the fewest resources, with the potential to exacerbate health inequities. Risks to privacy and perpetuation of bias are more likely to be avoided if AI technologies in public health are designed with knowledge of community engagement, existing health disparities, and strategies for improving equity. This viewpoint proposes a multifaceted approach to ensure safer and more effective integration of AI in public health with the following call to action: (1) include the basics of AI technology in public health training and professional development; (2) use a community engagement approach to co-design AI technologies in public health; and (3) introduce governance and best practice mechanisms that can guide the use of AI in public health to prevent or mitigate potential harms. These actions will support the application of AI to varied public health domains through a framework for more transparent, responsive, and equitable use of this evolving technology, augmenting the work of public health practitioners and researchers to improve health outcomes while minimizing risks and unintended consequences.

## Introduction

While public health has a rich history of innovation in areas such as vaccination, sanitation, and epidemiology [1], the field has been cautious in the widespread adoption of emerging technologies like generative artificial intelligence (GenAI). Calls for updated approaches to public health funding, programming, and research have been pervasive amid the changing landscape of health in the United States and globally [2]. The field of public health is at a crossroads, with growing recognition of the need for innovation to afford greater agility and responsiveness to emerging problems [3].

GenAI applications are expected to impact all sectors of society. Many public health practitioners and researchers are understandably cautious about these developments and how they will affect the discipline and the communities we serve—the exception is in bioinformatics and biostatistics, where machine learning and other techniques have long been applied to medical and health care data [4]. Given the speed of technology, there is a risk that significant portions of the population—particularly those with the fewest resources—will be marginalized by the incursion of GenAI technologies. An analogous example is the burden placed on individuals to use emerging technological tools for health care (eg, patient portals, telehealth visits, pharmacy apps), which disproportionately exclude low-income and aging populations. The present danger is that the AI revolution in public health may exacerbate well-known health disparities, both through new generative tools as well as common AI or informatics techniques being applied more broadly. As public health professionals, we must educate ourselves and engage communities in knowledge seeking around AI and its impact on their lives, to fulfill the goals of promoting and protecting the health of all people and their communities.

Adoption of technological innovations may be challenging, but working with communities has been a strength of the contemporary public health profession. The field has embraced community engagement and participation as a norm, in a way that can be leveraged to ensure that AI and all its applications are an innovation that benefits society and promotes health equity. Given that community-engaged design and participatory methods have become embedded in many public health projects [5], the field is primed for outreach to communities to ensure that individuals understand the benefits and risks of AI in public health. Community involvement in AI design and implementation is key for helping ensure that AI is developed in a person-centered way, particularly addressing the needs and desires of underrepresented and minoritized communities that often face greater health disparities [6]. Beyond health care applications, where tools have often been used for clinical decision-making, outcome prediction, and clinical record summarization, there are numerous use cases for AI in public health. Infectious disease surveillance and risk assessment have been a key area for the use of this technology [7,8]. The use of AI in global health has also been described for improving vaccine delivery and community health care efficiency [9], and as the digitalization of public health continues, the use of AI applications for monitoring and evaluation will become more frequent [10,11]. Chatbots developed with GenAI are another increasingly common approach to providing health education and behavior change support [12]. Large language models are being used to support question answering over large health datasets, allowing more informed decision-making [13]. There is also an urgent need to combat health-related misinformation and disinformation through understanding and using GenAI, as experts predict it could generate up to 90% of web-based content by 2026 [14].

But what about the ethics of using this technology when both public health professionals and people whose data are being used to build applications of AI do not have an understanding of how these systems work? Public health researchers, practitioners, and community partners will need to understand the basics of AI applications and use cases in public health, to be able to prevent and mitigate harm to fully realize the potential benefits of AI as a social innovation. Promoting the responsible and ethical use of AI in public health will require a proactive approach to learning that requires openness. Public health practitioners may view AI applications as the domain of technologists or informaticists only, but communities must be involved in codeveloping these technologies. The ethical implications of AI in public health necessitate transparency, accountability, and equity, all of which require the involvement of scholars, practitioners, and people with lived experience [15]. Education and awareness for communities and public health researchers are urgently needed.

While there are both real and perceived risks of privacy violation posed by using AI in health [16], another important potential harm is the risk of introducing or perpetuating biases and thus exacerbating existing health disparities. There are cases where such biases have led to individuals being denied access to health services or insurance, which may result in discrimination and violations of individual rights [17]. Statistical models are based on underlying assumptions, and these can have far-reaching consequences. Knowledge of community engagement, health inequalities, and strategies for improving equity are crucial to avoid inadvertent reinforcement of biases embedded in data. A well-known example is a racially biased single health care algorithm that drives important chronic care management for over 70 million people in the United States [18]. Because of significant racial bias in this widely used algorithm, White patients with the same health as Black patients were far more likely to be enrolled in a care management program and benefit from its resources. Public health domain knowledge in social determinants and structural inequities must be part of the developing uses of AI in population health science and practice.

## How the Public Health Community Can Take Action

For safer and more effective integration of AI in public health, a multifaceted approach should involve the following: (1) inclusion of the basics of AI technology in public health training and professional development; (2) community engagement for co-design of AI technologies applied to public health; and (3) introduction of governance and best practice mechanisms by public health organizations that can guide the use of AI in public health and prevent or mitigate potential harms.

First, public health programs and organizations should begin incorporating training on the fundamentals of AI technology into public health education and professional development. This will help ensure that public health professionals possess a foundational understanding of AI, allowing for critical assessment and effective use of AI tools in population health work. This training should not only cover the technical aspects of AI but also include a critical study of ethical considerations, known and unknown biases, and a component of the sociocultural implications of AI-driven decisions. Schools of public health are beginning to develop centers and training initiatives for this purpose, including the authors’ institutions, UNC Chapel Hill Gillings School of Global Public Health and Tulane University. A scoping review to map the literature from both public health and computer science databases is currently underway by the authors and will provide additional sources of training material for public health and AI education efforts. Furthermore, as a field, we must not only educate ourselves but ensure communities are informed about evolving AI use in public health and its impact on their lives by incorporating this into public health education.

Second, engagement and collaboration between technologists, public health professionals, and community members are vital in the development of AI technologies for public health. Involving community stakeholders from the outset will develop applications with a better understanding of the specific needs, desires, and values of the populations they are intended to serve [19]. Co-design processes can promote transparency, and when done responsibly and with care, can build trust, as they allow for the direct input of those who will be most affected by the technology [20,21]. An example of this is the partnership between the AFL-CIO and Carnegie Mellon University Human-Computer Interaction Institute, which engages workers in the innovation process around AI tools to address critical challenges and open pathways for collaboration. Hospitality workers partnered with university faculty to explore the potential impacts of AI in the daily execution of their jobs. Algorithmic managers, which focused on optimizing operations and the workflow of cleaning rooms, inadvertently increased work, creating potential health hazards, and decreased worker autonomy. In the partnership, stakeholders from the community, labor leaders, and academics engaged to mitigate potential health and occupational harms created by the application of AI [22]. This participatory approach, often a hallmark of contemporary public health approaches, enhances the relevance and acceptability of AI solutions that touch individuals and communities while also identifying and addressing potential biases or risks for disparity early in the development process.

Finally, the introduction of governance frameworks and best practice mechanisms by public health organizations is essential for guiding the responsible use of AI. These mechanisms should be designed to prevent or mitigate potential harms associated with AI, such as privacy violations, biased outcomes, or the exacerbation of health disparities. Governance structures should include clear guidelines on data management, algorithmic transparency, accountability, and ongoing monitoring and evaluation of AI applications. Additionally, the establishment of interdisciplinary oversight committees, including ethicists, data scientists, public health experts, and community members, can provide ongoing surveillance and ensure that AI tools are aligned with public health goals and ethical standards. One example is the National Institute of Health’s Artificial Intelligence/Machine Learning Consortium to Advance Health Equity and Researcher Diversity Ethics and Equity Workgroup. The Ethics and Equity Workgroup developed a set of ethics and equity principles, a glossary, and an interview guide. The ethical and equity principles comprise 5 core principles that articulate best practices for working with stakeholders from historically and presently underrepresented communities [23].

Together, these actions can potentiate the application of AI to varied public health domains, and likewise improve outcomes, through a framework for more transparent, responsive, and equitable use of this evolving technology, augmenting the work of public health practitioners and researchers to improve health outcomes while minimizing risks and unintended consequences. Without such safeguards, the application of AI technology in public health may continue to be dominated by technologists whose domain expertise does not include biomedical ethics training, a human rights framework, or an understanding of health equity and social determinants as they inform the central work of improving population health.

## Abbreviations

AI: artificial intelligence
GenAI: generative artificial intelligence
